# Humanized Klotho variants cause widespread transcriptional changes in B6 mice

**DOI:** 10.1002/alz70855_102892

**Published:** 2025-12-23

**Authors:** Anna L Tyler, Dylan Garceau, Kevin P Kotredes, Christoph Preuss, Michael Sasner, Gregory W Carter

**Affiliations:** ^1^ The Jackson Laboratory, Bar Harbor, ME, USA

## Abstract

**Background:**

The VS haplotype of the longevity factor klotho (KL) has been associated with decreased risk of Alzheimer's Disease (AD) and reduced AD brain pathology. To further explore the role of human variants of KL on AD pathology, we introduced the two human KL variants–the common FC variant, and the protective FC variant–into C57BL/6J mice.

**Method:**

We generated homozygous and heterozygous animals for each variant balanced with the wildtype mouse variant (FS). We measured whole brain gene expression at four months and 12 months and identified differentially expressed genes across mice carrying the different KL haplotypes.

**Result:**

At four months of age, there were no differentially expressed genes. However, at 12 months of age, there were 609 differentially expressed genes across the different haplotypes. These genes were enriched for synaptic function and protein translation at the synapse. Genes that were down‐regulated by the protective VS allele were enriched for synapse‐related terms, and those that were up‐regulated by the protective VS allele were enriched in ribosome‐ and translation‐related terms. In general, genes that were upregulated by the protective VS allele were downregulated by the common FC allele relative to the wild‐type mouse allele.

**Conclusion:**

These data suggest that the introduction of KL human variants to mice provides a translatable mechanistic model that will improve our understanding of the role of KL in aging and Alzheimer's Disease.

